# Supplementary material for: Positively charged specificity site in cyclin B1 is essential for mitotic fidelity
Source: Nat Commun. 2025 Jan 20;16:853. doi: 10.1038/s41467-024-55669-x (PMC11747444; doi:10.1038/s41467-024-55669-x)
Supplement: Supplementary file 1 — Supplementary Information [file 41467_2024_55669_MOESM1_ESM.pdf]

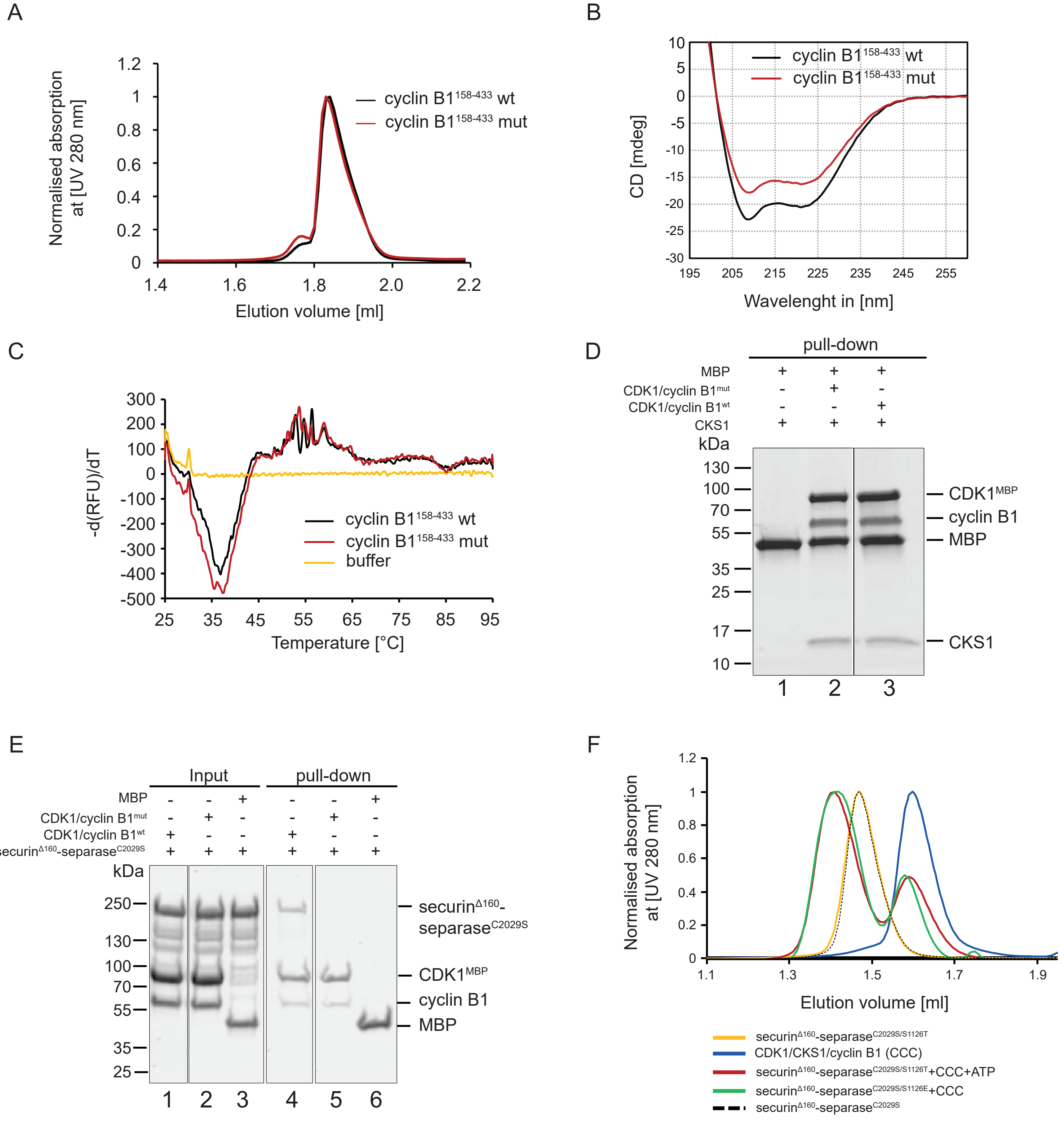
A
Normalised absorption at [UV 280 nm]
Elution volume [ml]
cyclin B1$^{158-433}$ wt
cyclin B1$^{158-433}$ mut
B
CD [mdeg]
Wavelenght in [nm]
cyclin B1$^{158-433}$ wt
cyclin B1$^{158-433}$ mut
C
-d(RFU)/dT
Temperature [°C]
cyclin B1$^{158-433}$ wt
cyclin B1$^{158-433}$ mut
buffer
D
pull-down
MBP
CDK1/cyclin B1^{mut}
CDK1/cyclin B1^{wt}
CKS1
kDa
CDK1^{MBP}
cyclin B1
MBP
CKS1
E
Input
pull-down
MBP
CDK1/cyclin B1^{mut}
CDK1/cyclin B1^{wt}
securin^{Δ160}-separase^{C2029S}
kDa
securin^{Δ160}-separase^{C2029S}
CDK1^{MBP}
cyclin B1
MBP
F
Normalised absorption at [UV 280 nm]
Elution volume [ml]
securin^{Δ160}-separase$^{C2029S/S1126T}$
CDK1/CKS1/cyclin B1 (CCC)
securin^{Δ160}-separase$^{C2029S/S1126T}$+CCC+ATP
securin^{Δ160}-separase$^{C2029S/S1126E}$+CCC
securin^{Δ160}-separase^{C2029S}

Supplementary Figure 1: Biochemical characterization of the phosphate-binding pocket of cyclin B1.

(A) Size exclusion chromatography (SEC) runs on untagged cyclin B1$^{158\text{-}433}$ wild-type (black) or pocket mutant (red) resulted in nearly identical elution volumes of approximately 1.83 ml, indicating that these complexes run as monomeric protomers with an identical fold. (B) Far UV circular dichroism (CD) spectra of cyclin B1$^{158\text{-}433}$ wild-type (black) or pocket mutant (red) at roughly 5 µM concentration. (C) Thermal-shift assays performed with cyclin B1$^{158\text{-}433}$ wild-type (black) or pocket mutant (red). The apparent melting temperatures are nearly identical at 36°C. (D) MBP pull-downs using recombinant CDK1 with an N-terminal MBP tag and C-terminal twin-strepII tag, cyclin B1$^{wt/mut}$ with a C-terminal 8xHis tag and purified CKS1. Both complexes pull down CKS1 efficiently. MBP served as negative control. (E) MBP pull-downs using recombinant MBP-tagged CDK1/cyclin B1$^{wt/mut}$ complexes with the same tags as described in panel (D) and purified securin$^{\Delta160}$-separaseC2029S. Only CDK1/cyclin B1^{wt} pulls down phosphorylated separase efficiently, whereas CDK1/cyclin B1^{mut} fails to pull down separase. MBP served as negative control. (F) SEC runs on the isolated securin$^{\Delta160}$-separaseC2029S (black dashed line), the isolated securin$^{\Delta160}$- separaseC2029S/S1126T (solid yellow line) and the CDK1/CKS1/cyclin B1 complex (solid blue line) resulted in elution volumes of approximately 1.46 ml, 1.46 ml and 1.6 ml, respectively. Adding ATP and the CDK1/CKS1/cyclin B1^{wt} complex to securin$^{\Delta160}$-separaseC2029S/S1126T results in a stable interaction between these two complexes (solid red line), due to phosphorylation of T1126 by CDK1. This is indicated by a clear shift towards higher molecular weight with an elution volume of roughly 1.4 ml. However, adding ATP is dispensable for complex formation between separase and the CCC complex in vitro when using a securin$^{\Delta160}$-separaseC2029S/S1126E mutant version (solid green line). Of note, in CCC complex CDK1 has a C-terminal 8xHis tag, cyclin B1 a C- terminal twin-strepII tag and CKS1 no tag.

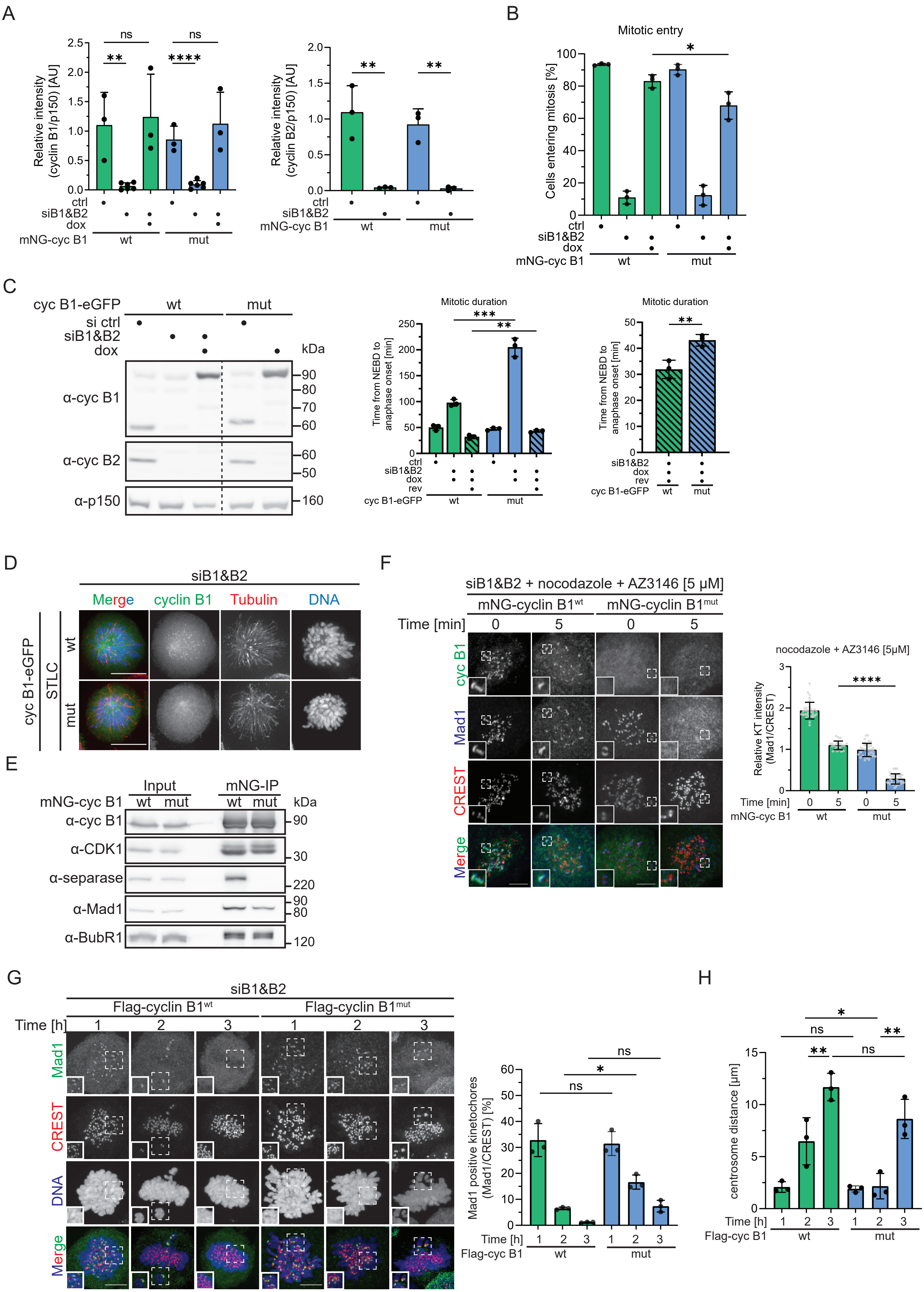
A
B
C
D
E
F
G
H
Relative intensity (cyclin B1/p150) [AU]
Relative intensity (cyclin B2/p150) [AU]
ns
**
****
ctrl
siB1&B2
dox
mNG-cyc B1
wt
mut
Mitotic entry
Cells entering mitosis [%]
cyc B1-eGFP
si ctrl
kDa
α-cyc B1
α-cyc B2
α-p150
Mitotic duration
Time from NEBD to anaphase onset [min]
rev
***
siB1&B2
Merge
cyclin B1
Tubulin
DNA
STLC
Input
mNG-IP
α-CDK1
α-separase
α-Mad1
α-BubR1
siB1&B2 + nocodazole + AZ3146 [5 μM]
mNG-cyclin B1wt
mNG-cyclin B1mut
Time [min]
cyc B1
Mad1
CREST
nocodazole + AZ3146 [5μM]
Relative KT intensity (Mad1/CREST)
Flag-cyclin B1wt
Flag-cyclin B1mut
Time [h]
Mad1 positive kinetochores (Mad1/CREST) [%]
Flag-cyc B1
centrosome distance [μm]
*

Supplementary Figure 2: Cyclin B1^{mut} causes mitotic defects.

(A) Quantification of WB shown in Fig. 2B. Left: Signal intensity of endogenous and ectopic mNG-tagged cyclin B1 in cells treated as indicated. Shown are mean of n=3 individual experiments. Significances calculated by unpaired t-test: **p=0.0018; ****p<0.0001. Right: Signal intensity of endogenous cyclin B2 in cells treated as indicated. Shown are mean/median of n=3 individual experiments. Unpaired t-test: **(wt)p=0.0079; **(mut)p=0.0022. (B) Quantification of mitotic entry from live cell movies. For details see schematic of Fig. 2A. Shown are mean values of n=3 individual experiments with indicated standard deviation. Unpaired t-test: *p=0.0499. (C) Quantification of live-cell imaging of cells stably expressing C-terminally tagged cyclin B1$^{wt/mut}$-eGFP. The mean of n=3 independent experiments is shown with standard deviation. Unpaired t-test. ***p=0.0006, **p=0.0096. Quantification shown in (C) for reversine conditions with different y-axis scale. Unpaired t-test **p=0.0096. Immunoblot assessing depletion of endogenous cyclin B1 and B2 and expression of cyclin B1$^{wt/mut}$-eGFP. p150 served as loading control. (D) Representative images of cyclin B1$^{wt/mut}$-eGFP expressing siB1&B2 cells arrested in prometaphase using STLC. Cyclin B1 (green), tubulin (red), DNA (blue). Scale bar = 10 μM. (E) WB assessing mNG-cyclin B1$^{wt/mut}$ IP samples from mitotically arrested cells depleted for endogenous cyclin B1 and B2. (F) Left: Immunofluorescence images of representative siB1&B2 cells arrested in mitosis using nocodazole (333 nM) expressing ectopic Flag-cyclin B1$^{wt/mut}$. Cells were forced out of mitosis using 5 μM AZ3146. Cells were fixed at indicated time points and imaged for cyclin B1 (green), Mad1 (blue) and CREST (red). Scale bar = 5 μm. Right: Quantification of the relative Mad1 intensity at the kinetochore. Significance is shown using one-way ANOVA: ****p≤0.0001. (G) Left: Immunofluorescence images of representative cells processed at indicated time points after release from STLC. For details see schematic of Fig. 2F. Mad1, CREST, and DNA are shown in green, red, and blue, respectively. Scale bar = 5 μm. Right: Quantification (left) of n=3 individual experiments. Significance is shown using one- way ANOVA: *p=0.0374. (H) Quantification of Fig. 2F. Significance is shown using one-way ANOVA: *p≤0.0279; **(wt) p≤0.0079; **(mut) p=0.0014.

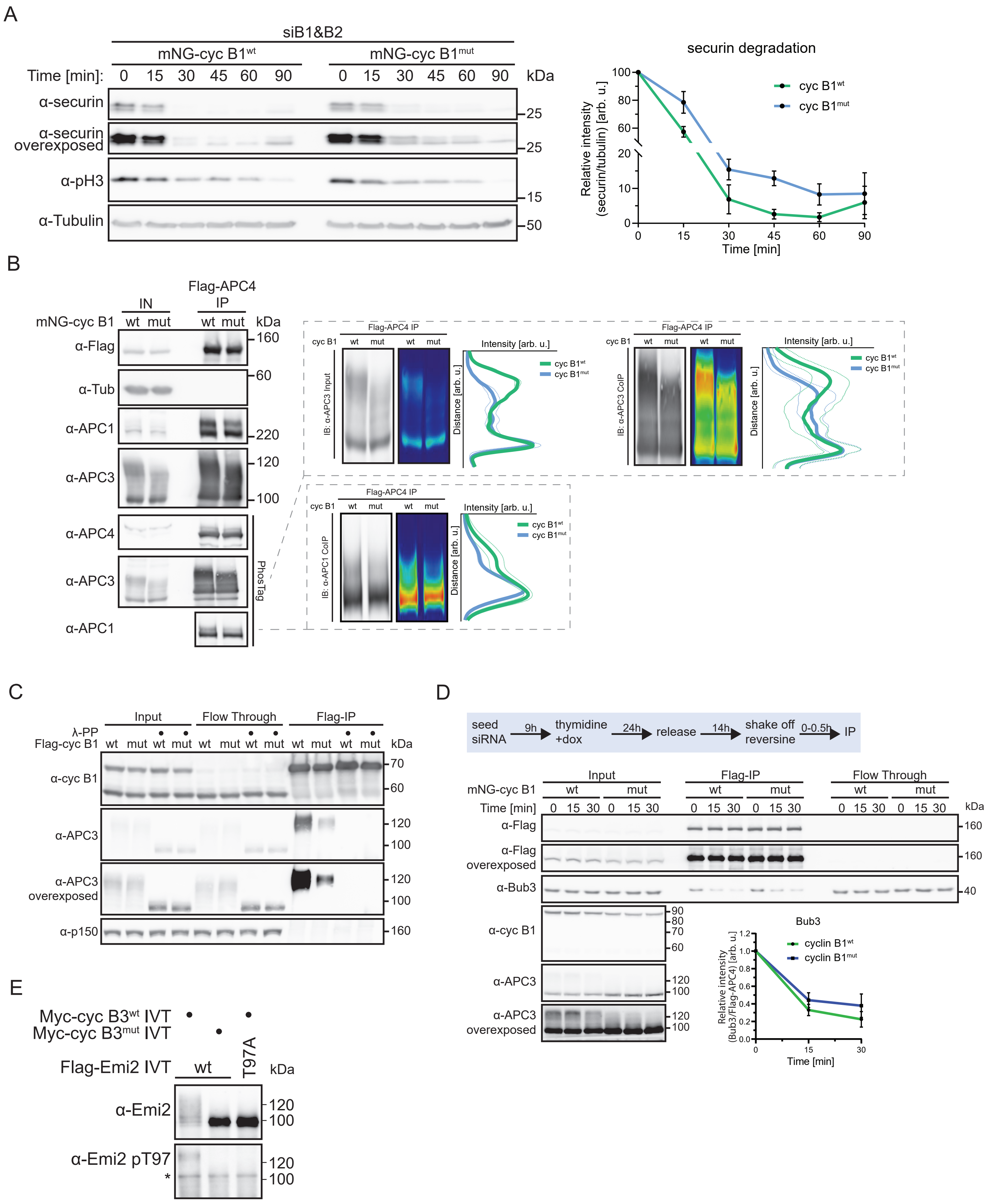

A
siB1&B2
mNG-cyc B1wt
mNG-cyc B1mut
Time [min]: 0 15 30 45 60 90 0 15 30 45 60 90 kDa
α-securin
α-securin overexposed
α-pH3
α-Tubulin
25
25
15
50
securin degradation
Relative intensity (securin/tubulin) [arb. u.]
cyc B1wt
cyc B1mut
Time [min]
B
Flag-APC4 IP
IN
mNG-cyc B1 wt mut wt mut kDa
α-Flag
α-Tub
α-APC1
α-APC3
α-APC4
α-APC3
α-APC1
PhosTag
160
60
220
120
100
cyc B1
IB: α-APC3 Input
IB: α-APC3 CoIP
IB: α-APC1 CoIP
Intensity [arb. u.]
Distance [arb. u.]
C
Input
Flow Through
Flag-IP
λ-PP
Flag-cyc B1 wt mut wt mut wt mut wt mut wt mut wt mut kDa
α-cyc B1
α-APC3
α-APC3 overexposed
α-p150
70
60
120
100
160
D
seed siRNA
9h
thymidine +dox
24h
release
14h
shake off reversine
0-0.5h
IP
Input
Flag-IP
Flow Through
mNG-cyc B1 wt mut wt mut wt mut
Time [min] 0 15 30 0 15 30 0 15 30 0 15 30 0 15 30 0 15 30 kDa
α-Flag
α-Flag overexposed
α-Bub3
α-cyc B1
α-APC3
α-APC3 overexposed
160
40
90
80
70
60
120
100
Bub3
Relative intensity (Bub3/Flag-APC4) [arb. u.]
cyclin B1wt
cyclin B1mut
Time [min]
E
Myc-cyc B3wt IVT
Myc-cyc B3mut IVT
Flag-Emi2 IVT wt T97A kDa
α-Emi2
α-Emi2 pT97
*
120
100

Supplementary Figure 3: Cyclin B1 interaction with APC/C is dependent on phosphorylations.

(A) Immunoblots of mitotic HeLa cells expressing mNG-cyclin B1$^{wt/mut}$. Cells were arrested in mitosis with nocodazole, and a SAC override was performed by the addition of MPS1 inhibitor reversine. Samples were taken at indicated timepoints. Quantification of the securin signal of n=3 independent experiments is shown with standard deviation. (B) Immunoblots of HeLa cell lysates mitotically arrested with nocodazole stably expressing Flag-APC4 and mNG- cyclin B1$^{wt/mut}$. Flag IPs were performed and immunoblotted against indicated proteins. PhosTag™ Gels were performed for APC4, APC3 and APC1. Quantification of the APC3 CoIP signal intensity is shown from n=3 independent repetitions and blotted against the distance on the blot. The same quantification is carried out for an APC3 CoIP from a mNG-cyclin B1$^{wt/mut}$ IP. (C) Immunoblots of mitotic HeLa cell extracts immunoprecipitated for Flag-cyclin B1. Cells were arrested in prometaphase by treatment with nocodazole and doxycycline for 20 h. Extracts were treated with or without Lambda phosphatase to broadly dephosphorylate cyclin B1 substrates. (D) Schematic used to synchronize siB1&B2 cells expressing Flag-APC4 in mitosis followed by shake-off and SAC inhibition for IP experiments. Immunoblots of Flag-APC4 IPs are shown for α-Flag, α-Bub3 α-cyc B1 and α-APC3. Samples taken at indicated timepoints. Quantification of CoIP'ed Bub3 signal from n=3 individual experiments. Mean value with standard deviation is shown. (E) Validation of the antibody raised against phosphorylated Emi2 T-97 (α-pT97). *Xenopus* egg extract was supplemented with IVT Myc-cyclin B3$^{wt/mut}$ (cyc B3$^{wt/mut}$) and full-length Flag-Emi2^{wt} or T97A. As indicated, samples were blotted against Emi2 and pT97-Emi2. Asterisk marks unspecific band.

Phosphopeptide/phosphosite analysis of APC/C *in vitro* phosphorylated by CDK1/CKS1/cyclin B1 (CCC^{wt}) or CDK1/CKS1/cyclin B1^{mut} (CCC^{mut})

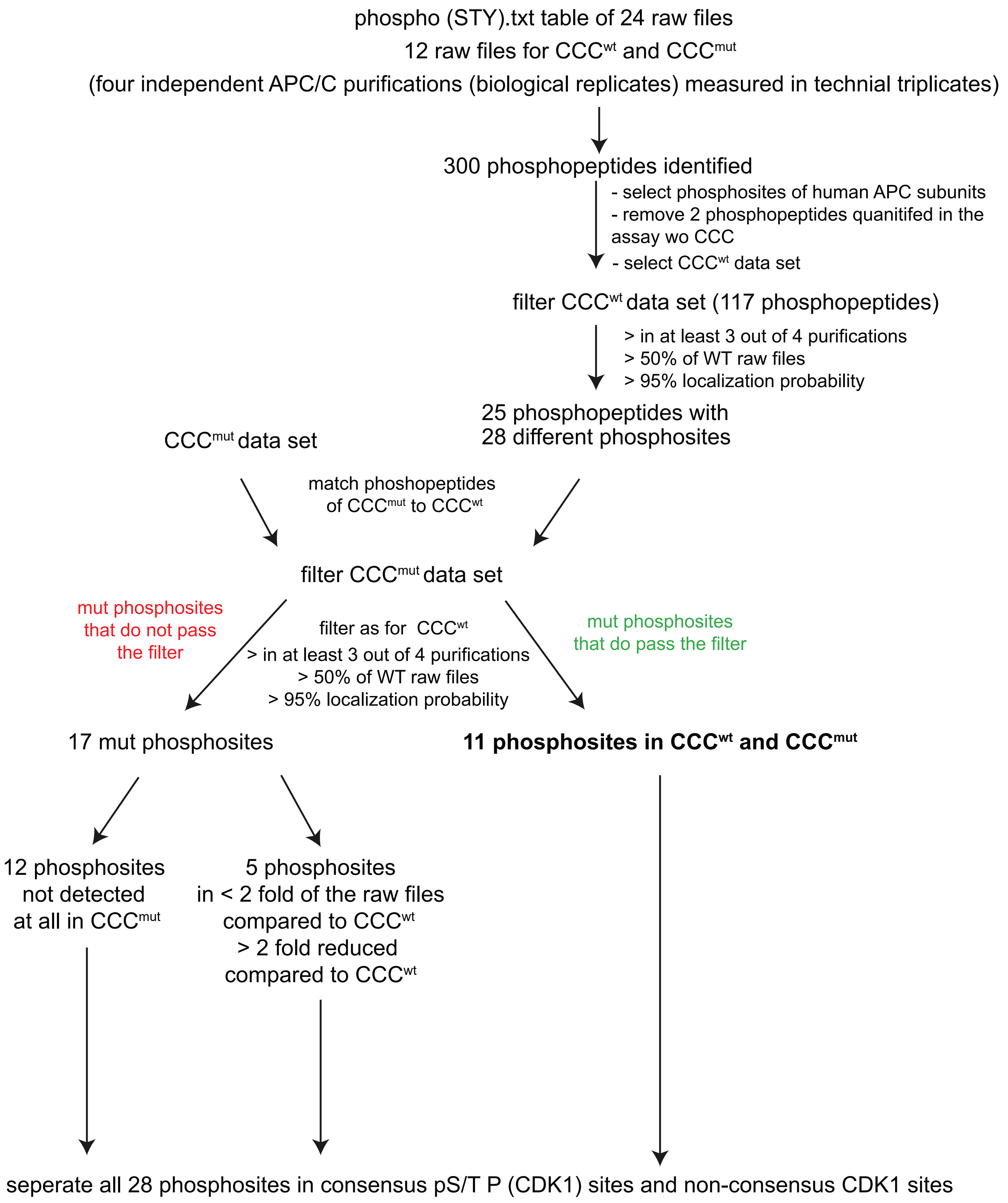

Supplementary Figure 4: In vitro phosphopeptide/site analysis.

Flow chart of the data analysis of in vitro phosphorylation assays using CCC^{wt} and CCC^{mut} to obtain a high-confident data set of phosphorylated sites which then has been used for the analysis shown in Fig. 4B.

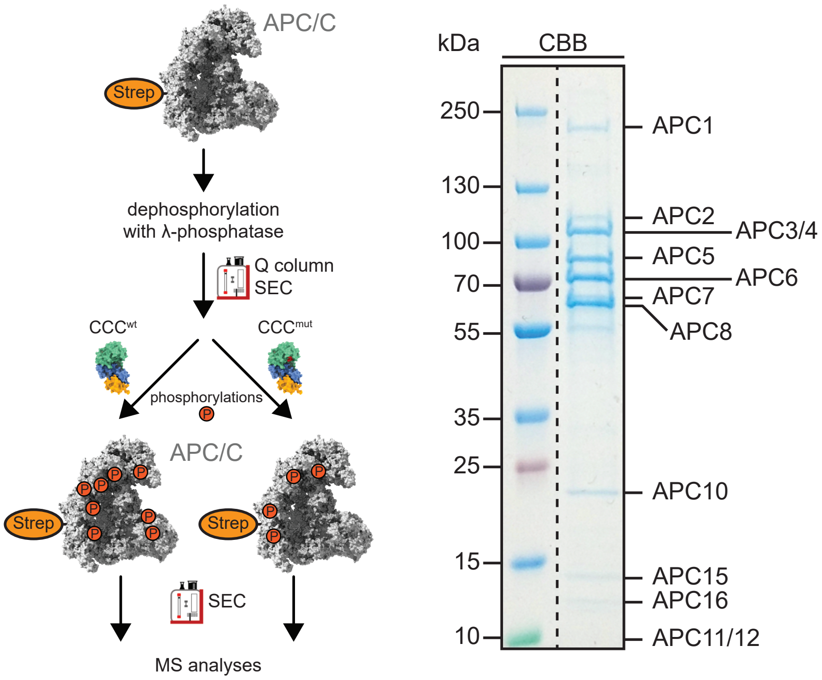

APC/C
Strep
dephosphorylation
with λ-phosphatase
Q column
SEC
CCCwt
CCCmut
phosphorylations
P
APC/C
Strep
Strep
SEC
MS analyses
kDa
CBB
250
130
100
70
55
35
25
15
10
APC1
APC2
APC3/4
APC5
APC6
APC7
APC8
APC10
APC15
APC16
APC11/12

Supplementary Figure 5: apoAPC/C purification schematic.

Experimental procedure of recombinant APC/C purification from insect cells. APC/C was dephosphorylated using purified λ-protein phosphatase followed by size exclusion chromatography (SEC) to remove phosphatase. Subsequently, APC/C was re-phosphorylated using recombinant CDK1/CKS1/cyclin B1$^{wt/mut}$ (CCC$^{wt/mut}$) followed by SEC to remove CCC. PDB 6TLJ and PDB 7NJ0 were used to illustrate APC/C and CCC, respectively. Coomassie brilliant blue (CBB) staining is shown of untreated purified APC/C with indicated subunits.
