# Supplementary material for: Positively charged specificity site in cyclin B1 is essential for mitotic fidelity
Source: Nat Commun. 2025 Jan 20;16:853. doi: 10.1038/s41467-024-55669-x (PMC11747444; doi:10.1038/s41467-024-55669-x)
Supplement: Supplementary file 2 — Description of Additional Supplementary Information [file 41467_2024_55669_MOESM2_ESM.docx]

**Description of Additional Supplementary Files**

File Name: Supplementary Data 1

Description: High-confident data set of in vitro APC/C phosphorylation assays using CCCwt and CCCmut
